# New distribution sites and local population trends of the white-eared night heron (*Gorsachiusmagnificus*) in China

**DOI:** 10.3897/BDJ.13.e159088

**Published:** 2025-06-12

**Authors:** Binqiang Li, Shanjun Ma, Nehafta Bibi, Miaodan Yang, Lifeng Tan

**Affiliations:** 1 Planing and Design Institute, Yunnan Forestry Technological College, Kunming, China Planing and Design Institute, Yunnan Forestry Technological College Kunming China; 2 College of Forestry, Southwest Forestry University, Kunming, China College of Forestry, Southwest Forestry University Kunming China; 3 Department of Zoology, Government Girls Degree College #1 Mansehra, Mansehra, Pakistan Department of Zoology, Government Girls Degree College #1 Mansehra Mansehra Pakistan; 4 College of Forestry, Guangxi Eco-engineering Vocational & Technical College, Liuzhou, China College of Forestry, Guangxi Eco-engineering Vocational & Technical College Liuzhou China

**Keywords:** The White-eared Night Heron (*Gorsachiusmagnificus*), endangered species, distribution range, population trends, Junwu Forest Park

## Abstract

The White-eared Night Heron (*Gorsachiusmagnificus*) is an endangered species facing increasing threats from human activities and ecological changes throughout its fragmented habitat. In this study, we integrate decade-long news data (2015–2024) and systematic field monitoring (2013–2024) to assess the species' distribution, local population trends and conservation challenges in China. Our analysis of 36 verified new reports revealed 14 new distribution sites across 11 provinces. Moreover, there were 17 reports of injuries to the White-eared Night Heron, including five wing injuries, four leg injuries, three eye injuries and five unspecified injuries. Our results suggest that discarded fishing lines, fishhooks and plastic contamination are the main causes of injuries to the species. For the local population, we recorded 12 breeding pairs of this species in Junwu Forest Park, Guangxi, China. However, our data showed a persistent decline in the number of adult individuals, nests and nestlings. The fragmented distribution pattern, combined with the threats it faces, means that, even if more and more of its distribution sites are detected, if adequate protective measures are not taken, its population may still face the danger of local extinction and further reduction. Our study highlights the role of social media data in assessing the population size and distribution of endangered species. From the perspective of species conservation, long-term monitoring programmes should be established in multiple sites across the species' range to provide a comprehensive understanding of population size trends and the effectiveness of conservation efforts.

## Introduction

The White-eared Night Heron (*Gorsachiusmagnificus*) is considered one of the rarest heron species worldwide, with extreme conservation urgency (Fellowes et al. 2001). Surveys from 1990 to 1998 documented its presence in merely six locations, highlighting its critically endangered status (Fellowes et al. 2001). From 1994 to 1996, the White-eared Night Heron was classified as Critically Endangered (CR) due to its very small population and fragmented habitat. Currently, its population size shows a declining trend (BirdLife International 2017). This decline is ongoing and is primarily caused by hunting and habitat destruction, largely driven by the demand for timber, agricultural land and illegal hunting (Liao et al. 2014, IUCN 2016).

The conservation status of *Gorsachiusmagnificus* has not been re-evaluated on the IUCN Red List of Threatened Species over the past ten years. This species was last assessed in 2016 (BirdLife International 2017) and is currently listed as Endangered (EN) under criterion C2a(i). In 2021, the Chinese government upgraded the protection status of *Gorsachiusmagnificus* from a national second-class protected species to a national first-class protected species to enhance its conservation efforts (http://www.forestry.gov.cn). Over the past two decades, an increasing number of distribution sites of *Gorsachiusmagnificus* have been documented (Zhao et al. 2006, He et al. 2007, Pilgrim et al. 2009, He et al. 2011, Zheng 2023, Jia et al. 2023). Previous studies indicate that *Gorsachiusmagnificus* is primarily distributed in the Chinese provinces (Shanxi, Inner Mongolia, Yunnan, Sichuan, Guizhou, Hubei, Hunan, Anhui, Jiangxi, Zhejiang, Fujian, Guangdong, Guangxi and Hainan) (Zheng 2023), as well as northern Vietnam (Pilgrim et al. 2009). More recently, new distribution records have emerged from India (Shafi et al. 2018), Bangladesh Sundarbans (Hossain et al. 2023) and Cambodia (Gray et al. 2019). The species may be considered for downlisting in the future if data show that the recent increase in its known range corresponds to a rise in the confirmed population (He et al. 2011, IUCN 2016, BirdLife International 2017).

The activities of the White-eared Night Heron are highly covert; it primarily flies and forages at night. This behaviour significantly reduces its detectability (Li et al. 2007, Gao et al. 2013, Jiang et al. 2017). For instance, during the breeding season, previous studies have demonstrated that adult birds leave the nest at night, with the peak departures occurring between 19:00 h and 20:00 h and return in the early morning, most frequently between 04:30 h and 05:30 h (Gao et al. 2013). After more than 20 years of field surveys and empirical studies by conservation biologists and ornithologists, we have a relatively clear idea of the geographic range and estimated population size, based on existing research and monitoring data of this species (Brazil 2009, BirdLife International 2017). However, to our knowledge, the distribution and population trends of this little-known species remain poorly understood. To understand the population trends of threatened species and the potential current and future risks, field survey data alone are not enough and multidimensional data and evidence are needed to develop a deeper and more comprehensive understanding (Wu et al. 2025). For example, social media data have been used to assess the population size, illegal hunting and trade of wildlife (Sardari et al. 2022, Salas-Picazo et al. 2023). Previous studies have shown that a well-supported social media community can provide valuable population data on elusive species in a specific area (Smith and Somers 2025).

In this study, we used news data from 2015 to 2024 to assess the distribution and threat factors of *Gorsachiusmagnificus*. We also report the trend of *Gorsachiusmagnificus* population size in Junwu Forest Park, Liuzhou, Guangxi Province, China. In fact, due to the limitations of funds and time, it is challenging for conservation biologists to assess the status of a threatened species in a short period of time. News data usually derive from citizen science and most reports are accidental observations. If such species data observed by chance are appraised by professionals, we believe that these data can be used to assess the threatened status of the species. By integrating news data and field survey data, we have provided potential data support for the conservation and threatened status of *Gorsachiusmagnificus*, which will serve as reference information for future assessments.

## Materials and Methods

### Study area

**Figure 1.**
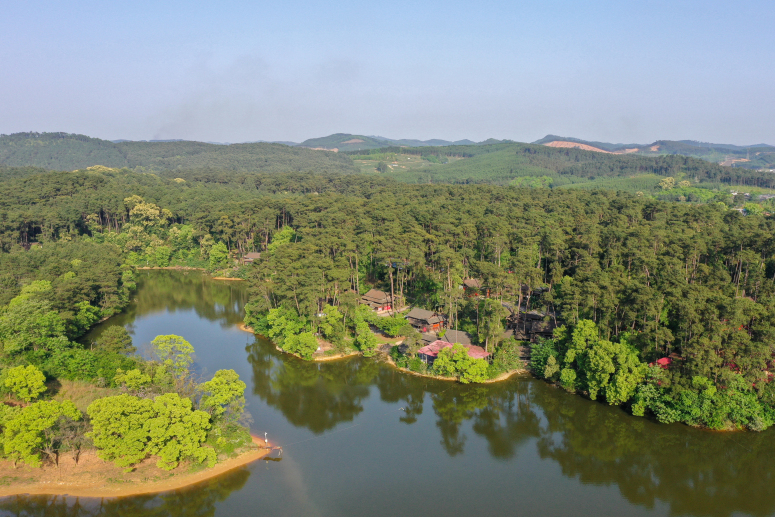
The White-eared Night Heron breeding habitat in Junwu Forest Park of Liuzhou, Guangxi, China.

To assess local population trends of the White-eared Night Heron, our fieldwork was conducted in Junwu Forest Park (Junwu, 24°27′21.59″-24°29′49.19″N, 109°20′16.8″-109°24′46.7″E; a total area of 338.8 ha), Liuzhou, Guangxi Province, China (Fig. 1). Junwu is located in the south subtropical monsoon climate zone, which is characterised by hot summers and mild winters (Tan et al. 2023). The mean annual temperature is 20.7°C and the region receives substantial precipitation, with an average of 1,535.6 mm annually (Tan et al. 2023). The vegetation is dominated by plantation conifers (e.g. *Pinusmassoniana*) and native plants (*Quercusgriffithii*, *Lithocarpusglaber*), with a dense understorey of *Clerodendrumcyrtophyllum* and *Maesajaponica* (Tan et al. 2023).

### Methods

We used China News Service (CNS, www.chinanews.com) and Baidu (www.baidu.com) to search for news reports of *Gorsachiusmagnificus* in China from 2015 to 2024. CNS is a state-level news agency in China providing news coverage to the world (www.chinanews.com). We used the following keywords for the search: "*Gorsachiusmagnificus*", "The White-eared Night Heron" and "Night heron". Furthermore, since the same data might be reported by multiple news agencies, we deleted the duplicate news data and retained only one of them. The data we extracted included the occurrence time, location and health status of this species. To assess the credibility of the news outlets and authors, we chose government news reports (such as the Bureau of Forestry and Grassland, the Protected Area Management Authority and the Wildlife Conservation and Rescue Center) and established and credible news publishers (such as China Central Television and local government-administered news broadcasters). For each reported sighting, we sought supporting evidence such as photographs, videos or audio recordings. Meanwhile, every news report contains at least one government administrator or ornithologist involved in identifying and managing the species. According to previous study reports (Fellowes et al. 2001, Zhao et al. 2006, He et al. 2007, Li et al. 2007, He et al. 2011, Gao et al. 2013, Zhong et al. 2014, Liao et al. 2014, Lu et al. 2016, Jiang et al. 2017, Yu et al. 2022, Zheng et al. 2023), a sighting documented in a region with no prior records of the species may require additional verification. In contrast, observations occurring within the species' established range and supported by previous records are more likely to be valid. Consequently, we reached out to ornithologists, conservation experts and local wildlife authorities for their opinions on the reported sightings. They may have additional information or insights that can help verify the authenticity of the reports (He et al. 2011).

**Figure 2.**
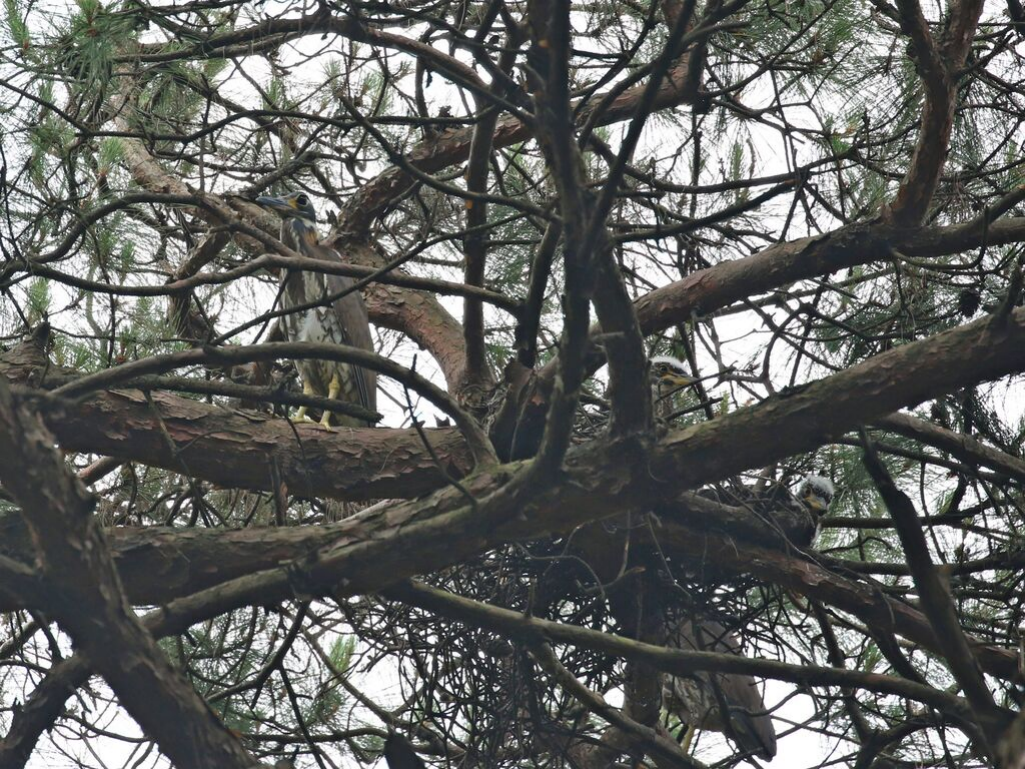
Adult and nestling of the White-eared Night Heron in Junwu Forest Park of Liuzhou, Guangxi, China.

To assess the population size trends of the White-eared Night Heron from 2013 to 2024 in Junwu, we conducted systematic surveys between February and August each year within the Park. We employed direct counting methods to estimate the population size. Once a nest was detected, we counted the number of adult birds and nestlings in each nest. For example, if we found the first nest contained two adult birds and three nestlings, we recorded a population count of five. If the second nest had two adult birds and two nestlings, we recorded a population count of four. Therefore, the combined population count for the first and second nests would be nine. The surveys involved actively searching for nest sites using telescopes and identifying faecal traces (Fig. 2). Our protocol ensured exhaustive spatial coverage, with repeated visits to all potential habitat sectors to minimise detection bias and validate population status. We used the Generalised Additive Models (GAMs) to analyse the population trend of the White-eared Night Heron. Population size was modelled using the Poisson distribution with a log-link function. All analyses were carried out in R software, the GAM being constructed using the mgcv package (Wood 2010).

## Results

### News data

**Figure 3.**
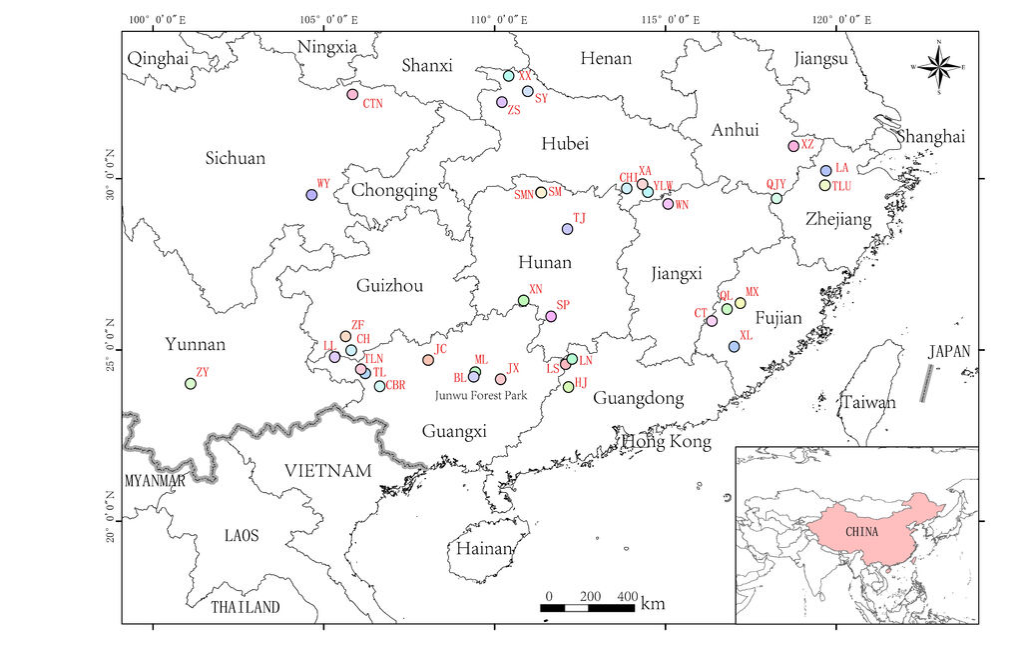
The distribution of the White-eared Night-heron with the localities mentioned in the news data (2015-204). Longlin LL; Tianlin TL; Wuning WN; Chibi CHI; Shimen SM; Zhushan ZS; Lianshan LS; Tonglu TLU; Xuanzhou XZ; Huaiji HJ; Lian'nan LN; Weiyuan WY; Shuangpai SP; Lin'an LA; Chengbi River CBR; Tianlin TLN; Zhenyuan ZY; Xinluo XL; Changting CT; Yueliang Bay YLW; Xian'an XA; Mingxi MX; Qingliu QL; Xinning XN; Xunxi XX; Chaotian CTN; Taojiang TJ; Qianjiangyuan QJY; Shimen SMN; Ceheng CH; Zhenfeng ZF; Malu Mountain ML; Bailian BL; Jinxiu JX; Jinchengjiang JC; Shiyan SY.

We collected data from 36 news articles published between 2015 and 2024 in China (Suppl. material 1). These include nine observations of the White-eared Night Heron reported solely by government managers and 27 observations reported by both villagers and government managers. The villagers handed the White-eared Night Heron individuals to the Forestry and Grassland Bureau and the Wildlife Rescue Center. According to news articles, the species was recorded in 11 provinces, including Guangxi, Jiangxi, Hubei, Hunan, Guangdong, Zhejiang, Anhui, Sichuan, Yunnan, Fujian and Guizhou (Fig. 3). Based on previous studies, Xianning, Changde, Shiyan, Qingyuan, Zhaoqing, Neijiang, Yongzhou, Lin'an, Pu'er, Longyan, Shaoyang, Yiyang, Kaihua and southwest Guizhou were identified as the new distribution sites of *Gorsachiusmagnificus* (Fig. 3, Suppl. material 1). Moreover, Jiuliangshan, Baise, Xuancheng, Neijiang, Xianning and southwest Guizhou are the breeding sites of the White-eared Night Heron (Suppl. material 1).

Importantly, there were seventeen reports of injuries to the White-eared Night Heron, of which five were wing injuries, four were leg injuries, three were eye injuries and five cases did not report the specific injured parts (Suppl. material 1). We noticed that only seven news articles identified the specific causes of the White-eared Night Heron injuries. Amongst these cases, three individuals were entangled in fishing lines, one was trapped in a plastic bag, one was caught in airport fog nets, one was involved in a car accident and one was associated with a hunting incident (Suppl. material 1).

### Local population

**Figure 4.**
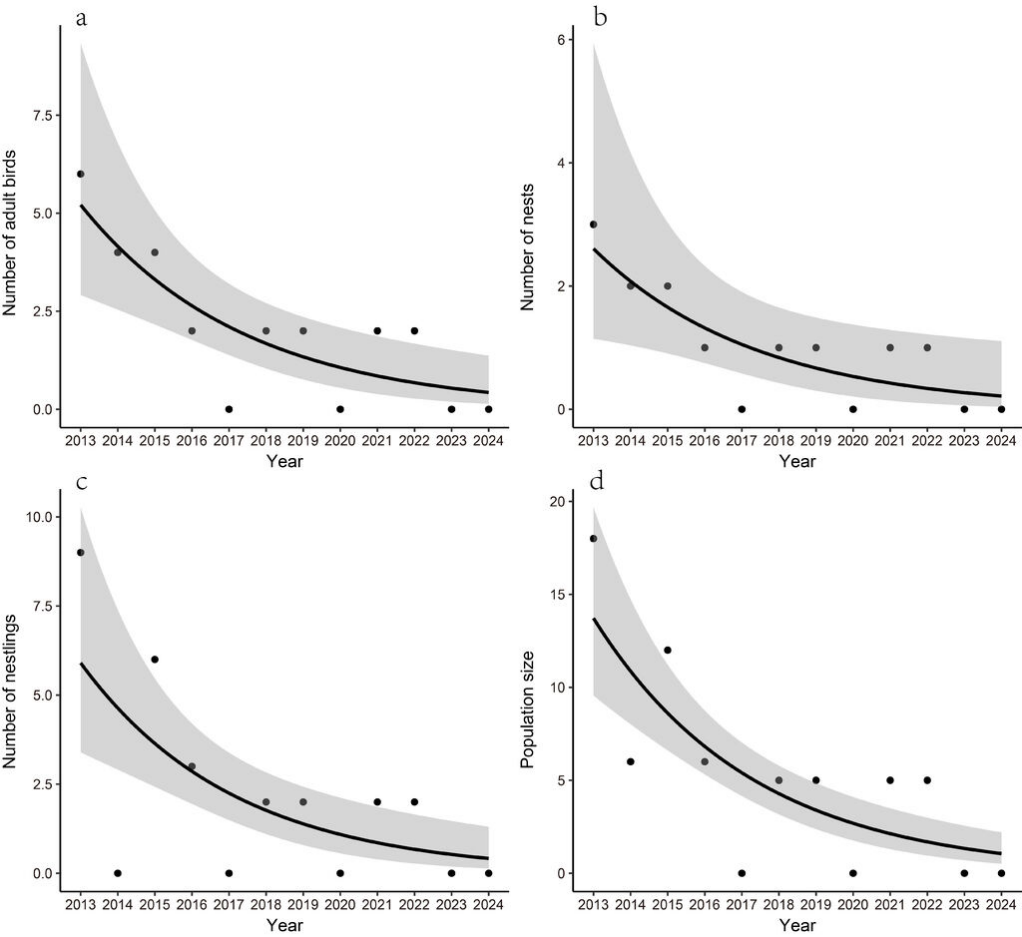
Population trends of the White-eared Night Heron in Junwu Forest Park, Liuzhou, Guangxi, China from 2013 to 2024. **a** number of adults; **b** number of nestlings; **c** number of nests; **d** population size.

Between 2013 and 2024, we recorded 12 pairs of the White-eared Night Heron breeding in Junwu. However, monitoring data from Junwu revealed a persistent decline in this species population (*p* < 0.001), manifested through decreasing counts of adult individuals (*p* = 0.001), active nests (*p* = 0.02) and nestlings (*p* < 0.001) (Fig. 4). Notably, breeding absence was recorded in four survey years (2017, 2020, 2023 and 2024), with no nesting activity or nestling production recorded during these periods. Additionally, in 2014, although adult individuals were observed, no nesting attempts or breeding behaviour were recorded.

## Discussion

### New distribution sites

Social media data have proven to be useful for assessing the distribution and threat status of wild animals. Furthermore, through public participation in scientific research, people's awareness and interest in wildlife research can be enhanced, thereby contributing to the development of more informed conservation strategies (Sardari et al. 2022, Smith and Somers 2025). Our identification of new distribution sites for the White-eared Night Heron across various provinces, as revealed by the news data, is both promising and worrisome. On the one hand, it suggests that previously overlooked populations are now being discovered. On the other hand, the scattered nature of these new sites and the fact that the species is still considered endangered underscore the fragility of the conservation outlook. We identified a total of six breeding sites. In fact, most of the new sites were recorded incidentally. It is not clear whether the species bred or overwintered there or whether they were resident birds. For example, previous studies proposed that Yunnan was a new distribution site for the White-eared Night Heron, but these data were mainly obtained, based on avian ringing records. Indeed, Xinping and Pu'er serve as transitional corridors within the species' migratory network rather than representing bona fide breeding or overwintering territories (Zhao et al. 2006, Jia et al. 2023).

Moreover, the presence of the species in areas with human activities, as evidenced by the news reports of injuries and the involvement of local villagers, highlights the complex interplay between human-wildlife interactions and the conservation of this species. These findings emphasise the necessity of developing a comprehensive understanding of the White-eared Night Heron's habitat fidelity and spatiotemporal ecology to provide information for targeted conservation strategies. For instance, the specific locations identified as new distribution sites could be prioritised for detailed ecological studies to determine the factors that support the presence of the White-eared Night Heron and to identify potential threats in these areas.

### Local population trends

Long-term monitoring and systematic surveys are important for understanding the White-eared Night Heron population dynamics and providing information for conservation actions. Previous studies suggested that the population of the White-eared Night Heron was historically limited. Brazil (2009) estimated fewer than 100 breeding pairs in China, with global mature individuals ranging between 250-999 (BirdLife International 2017). Recent findings suggest a significant underestimation of its population. A long-term monitoring study (2017-2020) in Qiandao Lake, Zhejiang, documented 310 breeding nests and 470 nestlings (Zheng et al. 2023). This regional dataset alone approaches the upper limits of previous global estimates, indicating that the actual population size of the species may require systematic re-assessment, especially in under-surveyed wetland and forest ecosystems throughout south-eastern China. However, the observed decline in the population of the White-eared Night Heron in Junwu over the study period is a cause for concern. The absence of breeding activity in certain years and the overall decrease in adult individuals, active nests and nestling counts indicate that the local population may be facing significant challenges. This decline could be influenced by a combination of factors, including habitat degradation, changes in environmental conditions and the cumulative impact of the threats mentioned earlier.

Since 2010, Junwu has undergone a significant landscape transformation driven by developments in tourism infrastructure, resulting in an increase in impervious surfaces. The expansion of cultural performances and motorsport events has contributed to a rise in annual visitor numbers, growing from 50,000 in 2010 to 410,000 by 2014 (Tan et al. 2023). Visitor numbers have since stabilised between 300,000 and 400,000 annually. Compounding these anthropogenic stressors, the ecosystem also faces heightened vulnerability to natural disturbances, including lightning-induced fires and biotic threats (Tan et al. 2023). Crucially, the invasion of *Bursaphelenchusxylophilus* has triggered mass mortality of *Pinusmassoniana*, the primary nesting substrate for the White-eared Night Heron (Zheng et al. 2023, Tan et al. 2023). These dead trees have been cleared by the government, potentially reducing the suitable habitat available for this species. Therefore, during the breeding period of the White-eared Night Heron, we recommend that the park authority should control the number of visitors, reduce motorcycle races and recreational parties. In addition, we also suggest strengthening the management of pests and diseases to prevent them from causing large-scale damage to forests.

### Challenges in conservation

The news data reports highlighting the injuries sustained by the White-eared Night Heron, including entanglement in fishing lines, entrapment in plastic bags, collisions with airport fog nets and road accidents, provide a stark reminder of the human-induced threats that this species faces. These threats are in addition to the ongoing issues of habitat destruction and hunting mentioned in the previous study (Fellowes et al. 2001, He et al. 2011, Liao et al. 2014). The fact that only a small proportion of the injury reports identified specific causes suggests that there might be many incidents which are undocumented and that the actual extent of human-related threats is likely much higher. The persistence of these threats, despite the species' protected status, underscores the challenges of enforcing conservation laws and regulations. Our findings suggest that conservation efforts should focus not only on habitat protection, but also on mitigating the direct impacts of human activities on the species. This could involve public awareness campaigns to educate local communities about the importance of the White-eared Night Heron and the need to avoid harmful interactions. Additionally, measures should be implemented to reduce the likelihood of such incidents, such as the proper disposal of fishing lines and other debris that could entangle the birds. China has not yet established designated protected areas specifically targeting the White-eared Night Heron conservation; only a small number of populations inhabit protected areas, such as Chebaling National Nature Reserve (Guangdong) (He et al. 2011, Gao et al. 2013) and Jiulianshan National Nature Reserve (Jiangxi) (Yu et al. 2022). For populations outside of protected areas, a coordinated conservation approach is necessary. This requires collaboration amongst researchers, local authorities and conservation organisations to develop and implement effective conservation plans.

## Supplementary Material

5ED0D3E1-80BC-590C-859C-07554D04F3B9 10.3897/BDJ.13.e159088.suppl1 Supplementary material 1 Distribution and threat status Data type Table Brief description Distribution and threat status of the White-eared Night Heron based on news data. File: oo_1314815.xlsx https://binary.pensoft.net/file/1314815 Binqiang Li
